# Money, Social Relationships and the Sense of Self: The Consequences of an Improved Financial Situation for Persons Suffering from Serious Mental Illness

**DOI:** 10.1007/s10597-017-0146-3

**Published:** 2017-05-11

**Authors:** Alain Topor, Ingemar Ljungqvist

**Affiliations:** 1 Department of Social Work, Stockholm University, Stockholm, Sweden; 2 Department of Psychosocial Health, Faculty of Health and Sports Sciences, University of Agder, Kristiansand, Norway; 3 Department of Education and Blekinge Centre of Competence, Linnaeus University, Växjö, Sweden

**Keywords:** Severe mental illness, Poverty, social network, Symptoms, Sense of self

## Abstract

During a 9-month period, 100 persons with SMI were given approx. 73 USD per month above their normal income. Sixteen of the subjects were interviewed. The interviews were analysed according to the methods of thematic analysis. The money was used for personal pleasure and to re-establish reciprocal relations to others. The ways in which different individuals used the money at their disposal impacted their sense of self through experiences of mastery, agency, reciprocity, recognition and security. The findings underline the importance of including social circumstances in our understanding of mental health problems, their trajectories and the recovery process.

## Introduction

Studies on the correlation between relative poverty (Lott and Bullock 2001) and severe mental illness (SMI) have identified a number of links between them (Read et al. 2013). People suffering from SMI are more likely to belong to lower socioeconomic groups than higher ones (Eaton 1980; Hollingshead and Readlich 1958). Poor people tend to be given more severe diagnoses, even when they exhibit the same symptoms as others who are better off (Read 2010). The treatment interventions they are subject to are more corporally oriented (hospitalization, medication, etc.), than those deployed for people from other social groups evidencing similar problems (Hollingshead and Readlich 1958; Johnstone 2000). Poverty may also hinder the propensity to recover (Mattsson et al. 2008).

Two opposing hypotheses have been advanced regarding the link between mental illness and social class—social drift and social causation. In the dominant social drift model, illness causes a downward shift in social class. Social isolation, apathy, lack of initiative and low sense of self are assumed to be symptoms of a biologically caused illness (Frith and Johnstone 2003) and the accompanying difficulties of holding down a job and managing personal finances have a pronounced detrimental effect on the life situation of the individual concerned.

The second hypothesis identifies relative poverty as a social causation of mental illness, through mediating factors such as the higher likelihood of poor people being subjected to various abuses and the stress and limitations that living in poverty entails (Draine 2013; Read et al. 2013).

These two hypotheses are not mutually exclusive (Read 2010; Yanos et al. 2010). Both assume mental illnesses and poverty as given conditions that can be assessed fairly unambiguously. However, some studies (Mills 2015), highlight the effect contextual factors have when it comes to deciding the behaviours to be selected as basis for diagnosis (Kirk and Kutchins 1992), and how these factors may influence which psychiatric diagnoses target different socio-cultural groups (Frances 2013; Read et al. 2013).

### Poverty and Social Isolation

People with SMI are described as having smaller social networks, where family plays a more prominent role, compared with the general population. Studies also pointed to a paradox—persons with SMI often express a longing for friendship but tend to remain socially isolated (Hansson et al. 2002). This might be interpreted as a sign of their inability and indifference to initiating, developing, and maintaining close relationships with others (Palumbo et al. 2015). One aspect of the connection between relative poverty and mental illness studied is the possibility that behaviour seen as symptomatic of SMI might be commonplace among poor sectors of the community (Cohen 1993).

#### From the Perspective of the Person

A few studies have examined the way in which poor people with SMI describe how they have been able to manage outside psychiatric institutions when faced with this double trouble. Ware and Goldfinger (1997), Wilton (2004), Caplan (2014) and Topor et al. (2014) reported similar findings in different socio-political contexts. The individuals they studied had developed both personal and collective ways of coping with financial constraints. These studies formulated a possible link between a life of relative poverty and some signs believed to be symptoms of SMI—apathy, social withdrawal, impoverished social networks and a negative sense of self (Davidson and Strauss 1992) marked by illness identity.

#### Changing Economic Preconditions

One randomized control study investigated this connection in greater detail (Davidson et al. 2001a, b, 2004). People with SMI who were deemed socially isolated received a financial contribution of $28 a month for a period of 9 months, to be spent on social activities. The results showed statistically significant improvements in functional level, the alleviation of some symptoms and an increased sense of self. Here, something usually understood as symptomatic of SMI was impacted by the individual´s financial situation.

Although the relationship between economic status and mental health is well established there is a dearth of studies that probe at any depth the impact of an improved financial situation on the wellbeing and mental health of persons with SMI, and as a result of their findings are able to develop new forms of helpful interventions.

The study carried out by Davidson et al. has been the starting point for the present study, which was conducted in a different socio-political context—Sweden, one of the Nordic welfare societies.

### The Swedish Intervention Study—The Quantitative Aspect

The overall aim of the study was to ascertain the possible impact of an improved financial situation on the symptoms, functional levels, social relationships, quality of life and sense of self of persons with SMI (Topor et al. 2016). The aim of this article is to present how the participants described the effects of their improved economic situation, 7 months in the interventions’ period.

A group of 100 individuals were recruited to the intervention group. A comparison group of 50 was asked to participate in the study, and 38 gave their informed consent. Inclusion criteria stipulated that participants were to be in ongoing contact with both local county psychiatric services and municipal social services, due to psychiatric disability. In the Swedish context it was assumed that this criterion was good-enough proxy for severe mental illness (Arvidsson 2008). This approach was chosen since recruitment was carried out via the social services, which do not have access to the persons’ psychiatric diagnoses. Participants in both groups were informed about the study both orally and in writing before they gave their informed consent to participating in the study.

The intervention consisted of a financial contribution of 500 Swedish crowns (approx. 73 USD or 53 EUR) per month during a period of 9 months during 2013 and 2014. The money was given to facilitate social activity and no control over how it was to be used was exercised.

Both groups were asked to participate at a number of assessments at the start of the project, 7 months into it, and 6 months after it had finished. The subjects in the comparison group received an allowance of 150 Swedish crowns (approx. 22 USD or 16 Euro) per evaluation occasion. The quantitative part of the study was complemented with a series of qualitative interviews (see below).

#### Results of the Quantitative Study

The assessments 7 months into the intervention period indicated a statistically significant easing of symptoms of depression and anxiety, increased satisfaction with respect to social interaction and an elevated quality of life and sense of self. No changes could be noted in the comparison group (Ljungqvist et al. 2015). Unlike the study conducted by Davidson et al (2001), no significant changes were observed in terms of functional level.

### The Qualitative Interview Study

The aim of the qualitative part of the study was designed to study how the participants used the money, and to gather the experience-based knowledge they acquired about the way this pecuniary intervention and their use of it influenced their social relationships, wellbeing and sense of self.

### Method

The interviews were collected in direct connection to the study, at the same intervals as the assessments.

The data presented in this article was collected on the second interview occasion, 7 months into the intervention period.

#### Recruitment to the Interview Study

To this end, we carried out a strategic selection among members of the intervention group. Sixteen individuals were contacted and informed about the qualitative study and asked to participate in three interviews. The selection of interviewees was made with the aim of creating as heterogeneous a group as possible in order to penetrate a wide variety of experiences (Glaser and Strauss 1967). The variables used to promote heterogeneity were age, sex, length of contact with the psychiatric services, residence in the city or in the countryside.

The interviewees consisted of 11 men and five women. Five were up to 34 years of age and 11 were between 35 and 65. Eleven lived in their own apartments or houses with the support of social services and five in supported housing. Ten lived in a city and six in the countryside. Six of them had a monthly disposable income (all form of income minus taxes) under SEK 10,000 (1460 USD). None had a disposable income over SEK 15,000 (2190 USD), which was the median for a single person in Sweden. Three were married or living together with a partner. One was divorced and 12 were single. They had a mean time of contact with psychiatric care of 20 years, median 18 years.

At the time of the study five participants were diagnosed as having schizophrenia, two some form of psychosis, four were bipolar, three suffered personality disorders and two anxiety syndrome.

#### The Interviews

The interviews were conducted at a location chosen by the interviewee; a cafe, the participant’s home, a municipal activity centre or the research unit’s premises. The interviews were recorded and transcribed in full and anonymized before being analysed by the two authors of this article.

The transcripts were analysed using thematic analysis methods described by Braun and Clarke (2008). The central aspect of this analytical approach is a moving back and forth between the transcripts and the emerging codes, themes and sub-themes. Although the study took its starting point in the work of Davidson et al. (2001a, b), we tried to implement an inductive approach, analysing the data at an explicit level. Both authors read all of the transcripts and ventilated patterns and general codes. These were presented to the interviewers and discussed with them. The first author then proceeded to carry on the analysis through constant comparison between empirical data and the provisory themes. Both authors discussed the themes that had been identified and the underlying data; the first author then further elaborated these themes. Both authors jointly carried out a concluding examination of the themes, their definitions and designations, and together selected the extracts that best illustrated these themes and sub-themes.

The study was approved by The Regional Ethics Committee in Lund. There are no known conflicts of interest.

## Results

The result section is divided into three parts corresponding to the three themes developed in the analytical phase. The first theme “The use of money”, dealt with how people sent their money. The second and third themes were based on the interviews from participants that experienced changes in their everyday lives that they traced back to the intervention. The second theme was “Money as a path to others and oneself” and is about how participants used money as a tool to (re)build relations with others and themselves. A third theme “Money and sense of self” focused on the impact the extra income and the particular way in which it was spent had on the sense of self.

### The Use of Money

The participants used the money in different ways, and it had different consequences for them. These consequences were linked to how they spent the money, and to where they were in their lives at that point.

It is possible to divide the interviewees into four groups according to the impact the intervention had on their daily lives, their social relationships and their sense of self.

#### More of the Same

Some of the participants let the money sink unnoticed into their personal economies. Mervin just did a little more of what he usually did: “It has been spent on both bad and good things ... I’ve bought cigarettes and a pizza or two and I gave some of it to my aunt.” The small positive consequences stemming from the intervention were preponderantly of an ephemeral nature. Mervin continues: “Collecting the money puts you in a good mood. It helps until there is none left. But when it’s gone ... then you have to start living normally again.” Mikael confirms this picture: “It just gets sucked up with the rest of your money, I think.”

Interviews with persons in this group do not suggest that the money in itself or as a tool transformed either their everyday lives or their sense of self. Most of them described themselves as satisfied with their social and psychological situation.

However, one person described an attempt to break out of his isolation. His experience shows the difficulty of breaking established patterns in relationships with others; Mark: “I wanted to use the money to invite my nephew to dinner, but I did not have to, because when I tried to get in touch with him, he never got back to me. He does not care about me at all” .

#### A Silver Lining

Several saw the money as an opportunity to temporarily improve their situation. They bought things for themselves or their home that they would not otherwise have been able to afford. Mats: “You feel good when you get the money. I have to say it’s been great having it. You know you have a little mad money. You can treat yourself to a pizza or a snack ... I spent some of the money on airplane and train models.”

Several describe it as a relief not to have to count the pennies, and to fear the month running up to the next instalment. Kajsa “Money is security, even when you only have a little of it. Knowing that you can make ends meet this month. Previously I borrowed, but I’ve always felt ashamed of doing so”.

The changes that the money brought about in their lives were described as enjoyable, but had no lasting impact on their social life, well-bring or sense of self.

#### Something is Happening

For others, not only the pecuniary intervention, but also the study itself showed that change was possible. In addition to enjoying the silver lining the money afforded some people took concrete steps to do something about their long-term personal finances.

I’ve stopped borrowing on the never-never, it only turns sour down the road. I realized it was time for me to break the pattern. I had debts in four different places, so a little of the money has been spent paying off debts. But the most important thing is that I have broken the pattern. (Max)

These changes may not be sweeping changes, but they give room for reflection and the realization that things may be starting to happen. Karin: “It’s been fantastic to be able to splash out on these luxuries and ... it really makes me feel much happier.” Here, a long-term plan suddenly seems possible. Karin continues:

I save a little all the time. A few hundred crowns a month. This gives you a margin if anything happens ... You always have that feeling you’re worth less than other people if you don’t have much money. Putting aside money gives you some security, but you also feel you are worth just as much as other people. It’s also about making your own decisions and choosing to save.

In this instance the money is not a means to the pleasures of things and relationships, but a tool that actually helps foster a better self-image.

#### Something Completely Different

Some of the participants characterize the study and the intervention as something of great importance to themselves. These individuals integrated the research project into their own personal agenda. The project seemed to have come during a particular phase in their lives when they were susceptible to ideas of change. “I have taken such a beating for such a long time, but I will use this money to get over to the other side ... the right side I mean” (Martin). The realization that the money opens up opportunities to be taken advantage of is also joined with practical ideas about ways in which it can be used to prolong the project.

I know it is only for a short time, so there is no point in raising my standard of living in any way, for ... then I will suddenly be five hundred short. (...) The advantage of buying furniture is that when the project is over and the trips just a memory, I can walk up to that bookcase and touch it ... I was worth this. And then I’ll fill it with good music and good books. It’s sort of like my own little church ... (Martin)

For several participants who recount a more fundamental change in their circumstances, involvement in the study sparked off a personal project:

It was a breakthrough, now it was bloody well up to me ... Five hundred might not seem that much, but ... I’ve taken myself by the scruff of the neck and ordered my finances and really stay within budget and get some quality of life in the long term. Now I’ve cut up my credit cards, and I’ve bought some new clothes. It was good to see that I haven’t lost my lust for life. (Kerstin)

In addition to the monetary aspect of the intervention the interview carried great significance for some of the people involved. They interpreted the thrust of the interview:

Things had gotten so bad I felt I was just a body that society wanted to stick on the substitute bench. You felt worthless. And all the things we knew despite everything, nobody was interested in that ... Well, then you start to think that you’re a has-been, but that’s absolutely not true. It’s the other way around, so now I feel I’m a resource. (Martin)

For them the interview was also an opportunity to reflect on their own lives and on themselves: “It was liberating to get it all off your chest, it just felt like, ‘No, now someone is listening to me and understands where I’m at…. Now I exist! ‘“(Martin).In combination with the intervention the interview was a significant vehicle of change. “It was perhaps when I talked to you the first time I got some of the zest to live back. Anyway things started to loosen up” (Kerstin).

### Money as a Path to Others and Oneself

Although for some the money was sucked into in their everyday economy without leaving any noticeable trace in their narratives, the subjects in the three other groups used the money for a variety of purposes that can be sorted under two different themes.

#### Money as a Path to Others

In the interviews it is apparent that prolonged lack of money adversely impacts relationships with others as well as damaging the perception of self, which also reinforces isolationist tendencies. Many of those taking part in the study established renewed contact with friends and family and intensified the contacts they already had.

Geographical distance and travel costs had been an obstacle that hindered interaction with friends and family members. Relative poverty is also seen as a cause of morbid introspection and social isolation. “It is much easier to be sociable and mix with people when you have some money. Otherwise, you brood. And you cut yourself off. You really do. You do not get out and about that much”.

But even when the individual concerned does not see a connection between financial woes and mental problems, the cash supplement can make a difference to people’s social life. Matheo:

I have started hanging out with old friends again; they understand that I have this problem. Now I know that if I go into town and spend time with people, I don’t have to worry. The money means that if I start feeling bad, I can always take the bus home. So it makes me feel safe...

The money is used here as a tool to create security in relation to both social interaction and mental problems.

Some material purchases have had an indirect effect on the social lives of the participants. One person bought a tablet and used social networks to develop new relationships and strengthen existing ones.

#### Treating Others

The money has not only been a tool to overcome geographical distances to others, but also the social distance that occurs when a person in a social interaction cannot reciprocate: “The other day I was able to buy my niece a present for her 20th birthday. It’s always more fun giving than getting. So it felt pretty good” (Karin).

The lack of reciprocity in relations with others is described as a major hindrance to interaction. The money enabled them to more firmly to establish themselves in rewarding roles such as that of grandmother:

I’ve been out playing with my grandchildren. And afterwards we had a bite of something really nice together and I bought things for the toddlers. It’s fun to give. Yes, you get a lot pleasure from that money ... It’s meant quite a lot to me. (Kristina)

The importance of being able to give may also help individuals come to terms with the guilt that often accompanies poverty: “I wanted to treat my daughter, give her something. I have thought a lot about how I was beset by black moods when I was single and poor and scrambling to look after two kids” (Kerstin).

Several participants used some of the money to buy things for the home to make it more enjoyable for themselves and somewhere where they could meet others. “I’ve become more sociable. People come around to my place and keep me company. We sit down and talk and drink coffee and make ourselves comfortable” (Katarina). The money has made it possible to invest in a variety of tools that are able to facilitate social intercourse.

#### Money as a Path to Oneself

Several chose to spend part of the money on things for themselves. Being able to treat yourself to something extra was seen as an important part of the project: “Every month I treat myself to something and that’s ... the icing on the cake, as it were.” (Kerstin).

More than one person stressed the importance of the feeling of wellbeing brought about by being able to indulge in something above the ordinary, despite its transient nature.

The money gives me the chance to do something I don’t get to do every month, spoil myself a bit. Even before the project got underway, I thought the five hundred extra crowns could make all the difference between having fun during the month and not having fun ... (Karin)

Many used the money to bring about a change in appearance. They chose to purchase and some of these clothes were of a practical nature, but often than not they were a way to make the person feel better.

Pretty much the first thing I bought was workout gear and a pair of running shoes—the ones I had were so dreary and worn out and I really like being able to get out and about; going for walks and things like that ... Yes, it’s because they’re clothes, when you’re a girl it’s so much to fun to smarten yourself up a bit. (Kerstin)

### Money and Sense of Self

Those people who experienced changes in their everyday lives and in their relationships with others as a result of the intervention, also recounted how these changes created a feeling of owning their own lives, and playing a role in the lives of others.

#### Mastery

The project allowed several participants to exercise increased control over their daily lives. In several cases the project had also meant they were able to better manage their spending and abstain from strategies that had proved to have had excessively negative consequences in the long run., such as running up a tab in the local store or exploiting credit cards, Kerstin: “Now I feel I’m the one who controls my economy and makes all the decisions”.

The extra money had impacted the intergenerational relationship, lessening the need to borrow or beg money from parents during the intervention.

In some cases, mastery even affected relations to psychiatric care, as people moved on with their own agenda and on their own initiative cut back on or even stopped taking their medication, as they felt it was not working or had far too many side effects. Finally, mastery is about one’s relationship to oneself and one’s own body. Karolin:

I’ve gone on a diet. (…) My life’s more fun now. Since we met seven months back I’ve lost eight or nine kilos. Nowadays I only eat salad. And I’ve stopped drinking completely; I drank a lot of beer previously. That’s where all the money went. So now I have some over. I’ve been teetotal ten weeks now.

#### Reciprocity

Reciprocity is about taking part in social relationships—not just as a beneficiary of other people’s help, support and care, but also as someone able to respond in the same vein. Reciprocity can be inviting friends home, but it can also be being able to treat children and grandchildren to various events and giving presents on anniversaries. The growth of reciprocal relationships is especially noticeable in informal social networks where people describe how they have recaptured roles over and above those of the “patient”. Karolin:

I spend all weekend with my children and grandchildren. And sometimes I babysit and look after them during the week. I haven’t done this before, but it’s not that difficult. I didn’t know if I could manage, but ... now I dare do it.

#### Own Pleasures

Most of the participants used the money to buy things for themselves. Some had been able do this even prior to the study, but the intervention made it possible for them either to do this more often, or to buy unnecessary things that would be excluded from a rational budget. To indulge oneself with something extra or smart was described as making oneself more worthy, not only in their own eyes but also those of others.

#### Recognition

The experience of reciprocity, pleasure and mastery was, for some participants, enhanced by the project itself, as a form of recognition that some of their problems were not self-inflicted, but a result of harsh life conditions and social-political rules and regulations. Martin:

I was tilting at windmills, and that made me feel really isolated. Now I have a project to lean against so I thought, well …shit. Let’s see if things take a turn for the better and they did that right away. (...). I actually think that when it comes ... that there is someone who sees me, who understands where I’m at… then I exist. Otherwise I was only one in the crowd, one of the statistics. This is more about me as a ... person. Here it’s me making the difference.

The interviews made it possible for them to formulate their experiences freely and to give voice to a narrative in which they were able to sustain life-projects of their own. The interviews were also seen as recognition of themselves as sources of knowledge.

#### Security

The money in itself had also a direct influence on the sense of self, even when it was not used to improve relationships or to modify other aspects of everyday life. Participants recounted that they put some of the money to one side to give them a margin when times took a turn for the worse. They described the building up of a nest egg and the ability to save as a way of creating a sense of security in the long run.

#### Everyday Life and the Sense of Self

Experiencing mastery, entering into reciprocal relationships, giving oneself pleasure and being a source of pleasure and not merely a cause for concern to family and friends, being recognized and acquiring a modicum of security—all these aspects impact the development of a sense of self as a person able to influence his or her own life and wellbeing. For some of the participants the money and the project fed into their own projects. For others it became an inspiration that seeded new projects. Klara:

I went into [town] to attend a funeral. I only had a five hundred note on me, it wasn’t enough, but I met a guy who lives there. So the funeral ... it wasn’t particularly sad for me, I just had fun when I was there and I stayed at his place. He’s been here to see me too.

In real life the project was influenced by the particular circumstances of the participants in ways that could not be foreseen.

## Discussion

A number of studies suggest that individuals suffering from SMI are only able to maintain small social networks (Palumbo et al. 2015) at the same time as they express a wish for greater social intercourse (Hansson et al. 2002). The incidence of a limited social network and the lack of initiatives to broaden it, despite a desire for more and deeper relationships have generally been interpreted as symptomatic of mental illness (Andreassen 1984; Davidson et al. 2004). The qualitative results of the Swedish study confirmed the findings of Davidson et al. (2001a, b).

The users’ experiences gathered in the present study help us understand the mechanisms behind the changes noted in the quantitative study. An analysis of the interviews shows a range of different pathways with some individuals describing little, small or no change and others reporting a significant impact on different aspects of their lives, and on their sense of self that can be associated to a recovery process (Mezzina et al. 2006; Tew et al. 2012).

The money became a source of gratification that improved the living conditions of the participants, and the quality of the relationships they enjoyed with their social network (Davidson et al. 2006).

### An Own Project

Persons with SMI deal with poverty in different ways (Caplan 2014), and if their economic conditions improve this makes it possible for them to deploy strategies that enhance their everyday lives and transform their longing for social intercourse into goal-directed practices in the form of personal projects.

The varying implications the intervention had for different individuals can be interpreted in the light of Strauss’s (1989) article on mediating processes in schizophrenia. Strauss suggested that the recovery process consists of a sequence: woodshedding, turning point and mountain climbing. During the woodshedding phase the person catalogues the resources and fragilities in his or her life and rolls out a change project on the basis of this inventory. An improved economic situation seemed to widen the range of opportunities available in this phase. Money may also serve as an incentive when implementing the envisioned projects and lead to the turning point. If the person was engaged in a turning point process, a better economy could serve as a confirmation that he or she was on the right track. “Mountain climbing” means that the person is in the process of achieving some of the goals set in his or her change project. In this phase, the money seemed to make it possible to secure the steps that the person has already taken “up the mountain” and opens up a vista over new steps to be taken.

The individuals who claimed that the money had not affected their daily lives, wellbeing or sense of self, seem to have been at a stage in their lives that they were relatively satisfied with. They did not see the financial contribution as a tool for change.

### Symptoms

The impact of money on “psychiatric symptoms” such as asociality, avolition, depression and anxiety seemed to be mediated through the individuals’ sense of self and the experience of mastery, reciprocity, security, recognition and the propensity to experience pleasure.

Pleasurable experiences and a sense of mastery are important aspects facilitating personal recovery (Davidson et al. 2006; Slade 2009). More unexpected is that these experiences seems also to mitigate the symptoms of mental illness, and contribute to a clinical recovery process (Slade 2009).

These results pose important questions: If a social intervention can have such consequences; what is the implication for our understanding of the causes and cures of phenomena classified as SMI (Priebe et al. 2013)? A contextual and social perspective would seem to be necessary to problematize a one-sided approach to behaviour that tends to be described exclusively in terms of the symptoms of an underlying mental illness. These phenomena may also be the expression of the reduced room for manoeuvre a life in poverty entails.

### Limitations

As for all qualitative studies the extent to which the results presented here can be generalized is uncertain. However, in this case the results are reinforced as they confirm the findings from an earlier, similar study (Davidson et al. 2001a, b).

Another limitation in this regard derives from the fact that this study has been carried out in one of the Nordic welfare states, where socio-economic conditions are likely to be significantly different from those in other countries.

It is also possible that the effects of the intervention may subside over time as it can be presumed that people will adjust to new economic levels. Here, the same conditions hold true as with other forms of compensation, such as salaries.

#### Reflexivity—Not Only the Intervention

It is apparent that research studies have a major impact on target subjects (Bourdieu and Wacquant 2007; Frances 2013). In the present study some interviewees themselves clarified this impact. The funds were handed out unconditionally; there was no control over the way in which they were to be used. Many saw the whole project and especially the interviews as a recognition of that they possessed valuable knowledge.

The interviews also helped them to articulate their experiences, to view these experiences and themselves from a distance, and enabled them to assemble the strands of their life stories in a coherent narrative. A thread that is often broken when a person suffers long-term problem that radically affect his or her life (Bury 1982).

It is clear that interviewing participants in a study can in itself be seen as an intervention that impacts the results of the study; the “Hawthorne effect” (Coombs and Smith 2003; Wikström and Bendix 2000). This should be borne in mind when any attempt is made to generalize our findings about the positive effect improved finances may have on the situation of persons with SMI. However, not all interviewees reported that their situation had improved, and the statistically significant changes noted in the quantitative study were relevant for the group as a whole, and not limited exclusively to the interviewees.

### Further Research

There is need for further research on the effect of pecuniary interventions on isolation, and, inter alia, the role personal finances play in the social life of individuals and for their well-being.
